# Halide Perovskites for Photoelectrochemical Water Splitting and CO_2_ Reduction: Challenges and Opportunities

**DOI:** 10.1021/acscatal.3c06040

**Published:** 2024-04-16

**Authors:** Krzysztof Bienkowski, Renata Solarska, Linh Trinh, Justyna Widera-Kalinowska, Basheer Al-Anesi, Maning Liu, G. Krishnamurthy Grandhi, Paola Vivo, Burcu Oral, Beyza Yılmaz, Ramazan Yıldırım

**Affiliations:** § Centre of New Technologies, University of Warsaw, P.O. Box Banacha 2c, 02-097 Warsaw, Poland; ¤ Department of Chemistry, Adelphi University, 1 South Avenue, Garden City, New York 11530, United States; † Hybrid Solar Cells, Faculty of Engineering and Natural Sciences, Tampere University, P.O. Box 541, FI-33014 Tampere, Finland; ‡ Chemical Engineering Department, Bogazici University, 34342 Bebek, Istanbul, Turkey

**Keywords:** photoelectrochemistry, halide perovskites, CO_2_ reduction, solar energy conversion, machine learning

## Abstract

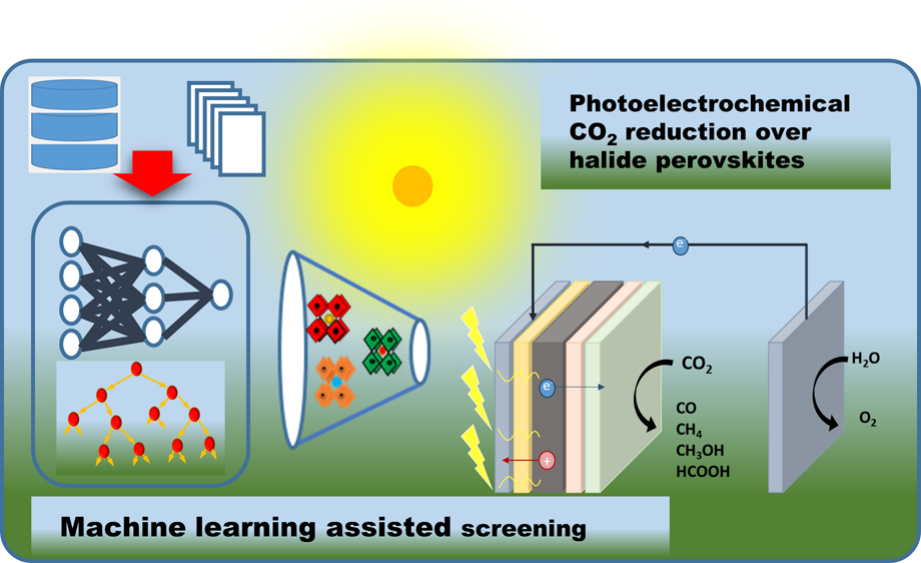

Photoelectrochemical water splitting and CO_2_ reduction provide an attractive route to produce solar fuels while reducing the level of CO_2_ emissions. Metal halide perovskites (MHPs) have been extensively studied for this purpose in recent years due to their suitable optoelectronic properties. In this review, we survey the recent achievements in the field. After a brief introduction to photoelectrochemical (PEC) processes, we discussed the properties, synthesis, and application of MHPs in this context. We also survey the state-of-the-art findings regarding significant achievements in performance, and developments in addressing the major challenges of toxicity and instability toward water. Efforts have been made to replace the toxic Pb with less toxic materials like Sn, Ge, Sb, and Bi. The stability toward water has been also improved by using various methods such as compositional engineering, 2D/3D perovskite structures, surface passivation, the use of protective layers, and encapsulation. In the last part, considering the experience gained in photovoltaic applications, we provided our perspective for the future challenges and opportunities. We place special emphasis on the improvement of stability as the major challenge and the potential contribution of machine learning to identify the most suitable formulation for halide perovskites with desired properties.

## 1 Introduction

Given the rapid depletion of fossil fuels and the global energy crisis, solar energy stands out as an attractive renewable energy source. Photocatalytic and photoelectrochemical processes such as water splitting and CO_2_ reduction provide an attractive route to utilize solar energy to produce H_2_, an energy carrier with great potential for the future, while reducing CO_2_ emissions. However, these processes require a catalyst, which is the most sensitive and challenging element in the whole value chain of the activation of small molecules such as H_2_O or CO_2_, involving the transfer of many electrons and protons. The development of stable photocatalytic structures that combine active site functionality, reaction rate, and intrinsic stability provides a gateway to multielectron activation and subsequent transformation of small molecules such as H_2_O or CO_2_.^1−6^ In addition, the electron structure dictating the charge transfer efficiency and the surface properties responsible for the activation of substrate molecules are crucial to the overall performance of the working system. Key shortcomings identified by recent studies include: *(i)* the inability of the materials to trigger and exploit the transfer of multiple charges to reactive sites, *(ii)* the inability to mimic the chemical environment more distant from the active site, which is important in controlling the access of reactants to the active site, *(iii)* the difficulty in lowering the free energy of the transition state, *(iv)* the inability to induce asymmetric charge distribution in a controlled manner to enhance the efficiency and selectivity of photoelectrochemical (PEC) water splitting and CO_2_ reduction processes. Metal halide perovskite (MHP) semiconductors, with their three-dimensional (3D) structure consisting of a [BX_6_] octahedral split at the corners, with A-cations occupying 12-fold cuboctahedral voids in the 3D lattice, and in particular, the associated high degree of symmetry, have demonstrated exceptional optoelectronic properties.^7−16^ These include, but are not limited to, high absorption coefficient, band tunability across the visible spectrum (400–800 nm), high carrier conductivity (1–10 cm^2^ V^–1^ s^–1^), long carrier lifetimes, and long charge diffusion lengths (∼1 μm).^17−20^ Such a unique combination of intrinsic properties of MHP semiconductors has led to the development of promising optoelectronic devices, such as photovoltaics, light emitting diodes, lasers, and photodetectors. More recently, MHPs have also been used in photocatalytic applications.

Halide perovskites, thanks to their 3D structure and a crystal lattice flexible to multiple structural modifications, can enable efficient transport of charges in the bulk and on the surface providing an alternative solution toward efficient and rapid water splitting as well as CO_2_ reduction. However, the stability of MHPs, especially toward water, which is the key issue in terms of long-lasting and efficient PEC processes, is a serious concern. Various approaches, such as the use of more stable components (especially cations) and structures (for example, monocrystalline structure), surface passivation, or encapsulations, have been employed to improve the stability of MHPs, often affecting, the MHPs’ toxicity and band gap simultaneously. Consequently, as reviewed below, numerous works on photoelectrochemical application of halide perovskites have been reported in various papers in recent years, while some review papers covering various aspects of the subject have been also published. For example, Shyamal et al.^21^ reviewed the photocatalytic CO_2_ reduction over halide perovskite nanocrystals, and Chen et al.^22^ reviewed the applications of these materials in both in water splitting and CO_2_ reduction in 2020. Similar works have been continued to be published in recent years covering CO_2_ reduction^23^ or water splitting and CO_2_ reduction^24^ together. In one of the more recent works, Wang et al.^25^ extensively reviewed the synthesis and use of halide perovskites for CO_2_ reduction while Chen et al.^26^ discussed the strategies to improve the stability and photocatalytic activity of halide perovskites.

In addition to the experimental works in the field, machine learning (ML) has also been used extensively in recent years for accelerating the discovery of new MHPs with desirable properties (especially stability and band gap) for visible light harvesting. Density functional theory (DFT) simulations (sometimes together with ML) have also been used to identify novel MHP compositions and understand their optoelectronic properties. The ML screening of DFT generated MHPs data has also been extensively employed in recent years. The accumulation of a large amount of data/knowledge on these materials due to their widespread use in photovoltaics since the 2010s, the presence of a large number of alternative structures that can be formed by changing the anion, cation, and their ratios, and the need for stable and safe but still sufficiently efficient structures made ML/DFT-assisted screening an effective route to discovering new halide perovskites with improved properties. Consequently, numerous research papers, including several reviews, have been published on ML applications of halide perovskites. Although most of the works published so far have focused on halide perovskites solar cells, the experience gained in the field will be also beneficial for PEC applications as well because the visible light harvesting efficiency and stability are also the major performance measures in PEC applications, which have also been presented in the scientific literature more frequently in recent years due to the progress achieved in the water resistance of halide perovskites.^27−30^

The scope of this review is to discuss the current state-of-the-art metal halide perovskites in relation to the key limitations of PEC water splitting and photoelectrochemical CO_2_ reduction, which need to be addressed in order to achieve substantial efficiency improvements for these processes. Herein, we present a critical assessment of the application of various MHP semiconductors, the challenges related to their poor stability, and future opportunities leveraging the massive experience in MHP photovoltaics. We think that our work is novel in several aspects and will make significant contribution to the field. We covered all relevant issues of the subject, including the synthesis of halide perovskites and their utilization in photoelectrochemical water splitting and CO_2_ reduction, while especially focusing on the recent developments to improve the stability of halide perovskites (especially against water), which is a major challenge in utilization of these materials in photoelectrochemical processes. Another important characteristic of halide perovskites is that they can be in large variety of configurations considering the large number of alternatives for A, B, and X sites. However, the number of synthesizable and stable structures with suitable electronic properties is limited; hence, the use of machine learning to discover or design new halide perovskite structures constitutes an important part of efforts toward the effective use of halide perovskites in photoelectrochemical reactions. Consequently, as different from similar reviews in the literature, we also covered the ML applications in the field and provided a future perspective for the utilization of this important tool in photoelectrochemical applications of halide perovskites.

## 2 Solar Driven Processes: Current State of Art in PEC Water Splitting and CO_2_ Reduction

### 2.1 PEC Water Splitting

As of today, various semiconducting materials such as metal oxides, metal sulfides, and metal nitrides including TiO_2_,^31−33^ WO_3_,^34,35^ Cu_2_O,^36,37^ CuO,^38^ BiVO_4_,^39,40^ Fe_2_O_3_,^41,42^ CdSe,^43^ CdS,^44,45^ Ta_3_N_5_,^46^ and C_3_N_4_^47−49^ have been investigated for water splitting in PEC cells. However, the photocatalytic activity of these materials is often limited due to the sluggish kinetics of oxygen evolution or too positive potential of the conduction band to drive hydrogen evolution. The reaction of oxygen formation through water oxidation, which is a necessary semireaction toward hydrogen realization, is thermodynamically and kinetically demanding. Consequently, a small number of electrons can react in the reduction reaction to form H_2_ (2H^+^ + 2e^–^ → H_2_), due to the recombination of photogenerated h^+^ and e^–^; only a small number of these electrons would be able to reach the semiconductor surface, where the reaction takes place. The band gap energy of the conduction band (CB) should be more negative than the potential for the hydrogen evolution reaction (HER) (0 eV) and the band gap position of the valence band (VB) more positive than that for the oxygen evolution reaction (OER) (1.23 eV), and although they can produce hydrogen and oxygen, they can also react with water causing back reactions, which in turn reduces the overall efficiency of the photocatalytic process. Finally, semiconductor materials often suffer from poor absorption of visible light due to their band gap width and electronic structure. Furthermore, the efficiency of the solar-to-hydrogen/hydrogen-to-hydrogen (STH) conversion process is highly dependent on the chemical stability of the semiconductors, which can be affected by many factors, such as pH, halogen introduction, interference ions, and so on. Various strategies have been proposed, including the use of new and more efficient semiconductors like MHPs, heterojunction design through the use of a cocatalyst on the semiconductors to improve light-harvesting efficiency, promote the charge separation process, and provide a protective layer for the underlying substrate materials.

The most important aspect of efficient charge separation and transfer from the semiconductor to the cocatalyst is the engineering of a suitable semiconductor/cocatalyst heterojunction.^50^ In addition, cocatalysts can provide a lower overpotential for the reduction of water molecules compared to photocatalysts.^48^ Furthermore, appropriate band alignment of the formed heterojunctions can improve visible light harvesting.^51^ Historically, water oxidation catalysts have been based on noble metal materials as the large-scale implementation has been hampered by the limited availability and cost of these metals.^52^ Hence, the development of nonprecious and metal-free materials is essential. Low-cost and earth-occurring transition metals such as Cu,^53−57^ Ni,^58^ and Co^59,60^ are examples of nonprecious elements that have been successfully used as cocatalysts in HER. Carbon-based supports such as graphitic carbon nitride (g-C_3_N_4_), graphene and its derivatives,^61−63^ as well as an organohydride-catalyst^64^ may also enhance photocatalytic CO_2_ reduction performance by overcoming the limitation faced by a traditional photocatalyst through their suitable physiochemical and electrical properties like high surface area, stability, anticorrosion capacity, photosensitivity, and conductivity.

While there are many solutions and approaches to overcome the slow oxygen formation in PEC water splitting, one common problem that needs consideration lies in multielectron reactions and the need for long-lived holes on the surface.^65^ The latter is determined by the efficient transport of charges in the bulk and on the surface, which is why halide perovskites, thanks to a crystal lattice flexible to multiple structural modifications, can provide an alternative solution toward efficient and rapid water splitting.

In summary, the goal is not to find a semiconductor that can do both CO_2_ reduction and O_2_ evolution but to find complementary light absorbers for both reactions. As we reviewed in Section 3, MHPs may be the potential solution for low efficiency of the PEC water splitting process even though they have their own challenges to overcome as we summarized in Section 4.

### 2.2 Photoelectrochemical (PEC) CO_2_ Reduction

**Figure 1:**
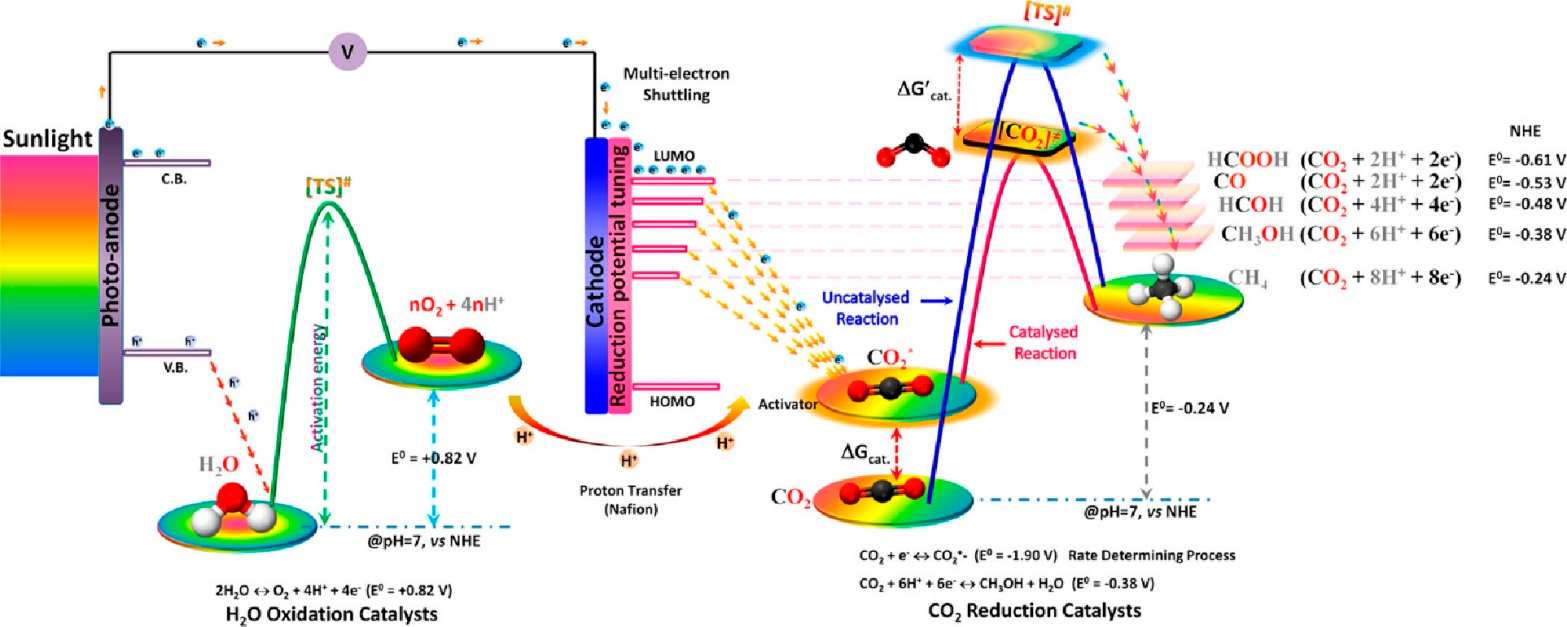
Schematics illustrating the band gap energetics involved in the water oxidation and CO_2_ reduction reactions in PEC and the role of the activation catalyst in thermodynamic and kinetic terms. The symbols “*” and “‡” represent the activated state and the transition state of CO_2_ by adsorption on the cathode surface, respectively. Reproduced with permission from ref (68). Copyright 2019 American Chemical Society.

PEC CO_2_ reduction has the following advantages over other electrochemical approaches: *(i)* significantly reduced overpotential required for uphill processes, *(ii)* relatively low cost of cell construction and operation, *(iii)* modulated, through applied potential, product selectivity and distribution, *(iv)* tuned band gap energetics that are useful parameters to help reduce CO_2_ activation energy. However, before running any reduction system, a key question must be answered: how does one activate the inert CO_2_ molecule? Figure 1 illustrates a schematic of the thermodynamic energy levels required to be overcome prior to the start of the water splitting or CO_2_ reduction reaction in a PEC cell, in the presence and absence of a catalyst. The green curve is assigned to the activation energy required for the water splitting reaction and illustrates the oxidation reaction of water with proton production passing from the H_2_O to the O_2_ energy level. The red and blue lines are for the CO_2_ reduction reaction and represent the activation energy curves in the presence or absence of catalysts, respectively. As can be seen in the diagram, electron transfer can help reduce the activation energy of the CO_2_ reduction reaction if a suitable electron transfer accelerator (e.g., a metal plasmon) is used.^66^ The overall CO_2_ reduction reaction can be achieved by combining electrons with appropriate energy levels, which are necessary for the activation and subsequent reduction of CO_2_. To obtain better product selectivity, CB and VB positions of the photocatalyst should be fully aligned. The major difficulty is to find a pristine semiconductor material with the right band gap energy position to suit both processes, namely the CO_2_ reduction and the water oxidation reaction.^67^

Although great progress has already been made, there are still areas of concern for CO_2_ reduction in PEC cells, such as *(i)* insufficient light absorption, *(ii)* inefficient charge separation and transport, and *(iii)* slow catalytic conversion at the surface. There are two main factors affecting the extent of light absorption by a photocatalytic material; the bandwidth and surface properties of the semiconductor materials used. Planar semiconductors show a relatively low light absorption efficiency due to their high surface reflectance. In general, inadequate light absorption is the main problem responsible for the reduction in total energy conversion efficiency, creating a need for new semiconductors like MHPs with high visible light harvesting capabilities. In addition, the total energy conversion efficiency is even more negatively affected by strong recombination processes of photoexcited charge carriers due to surface states, impurities or defects.^69^ The quality of the interface and its electron structure affect the transport of minority carriers toward the surface, which can be inhibited by a weak embedded electric field. Last but not least, surface catalytic conversion is challenging due to the complex electrochemistry of CO_2_ reduction. Due to the two stable, difficult-to-activate and dissociate C=O bonds present in the CO_2_ molecule, a large overpotential is required to overcome the CO_2_ reduction reaction barrier, as observed in the slow electrochemical kinetics of CO_2_ reduction compared to the competitive HER.^70−72^ Furthermore, CO_2_ reduction can occur via several possible reaction pathways, resulting in a low reaction selectivity. The introduction of suitable cocatalysts could increase activity and improve conversion selectivity.

Although noble-metal-based cocatalysts are the most widely used and can help achieve high CO_2_ conversion efficiencies (CO being the main product), as they do in water splitting, their rarity and high cost severely limit their large-scale use. However, highly abundant materials such as Cu-based catalysts used in PEC-based CO_2_ reduction for hydrocarbons and alcohols do not offer good product selectivity. Even though the use of biological cocatalysts avoids side reactions and gives high selectivity for a specific product, their large-scale use is also limited due to their high cost and poor stability under light irradiation. Reports on the use of carbonaceous materials in CO_2_ reduction by PEC processes are rare. Yet, there are very promising carbonaceous candidates as ideal cocatalysts. Their unique surface configurations and large active surfaces facilitate efficient adsorption and activation of CO_2_ molecules yielding high CO_2_ reduction efficiencies.^73^

## 3 Halide Perovskites

### 3.1 General Overview

**Figure 2:**
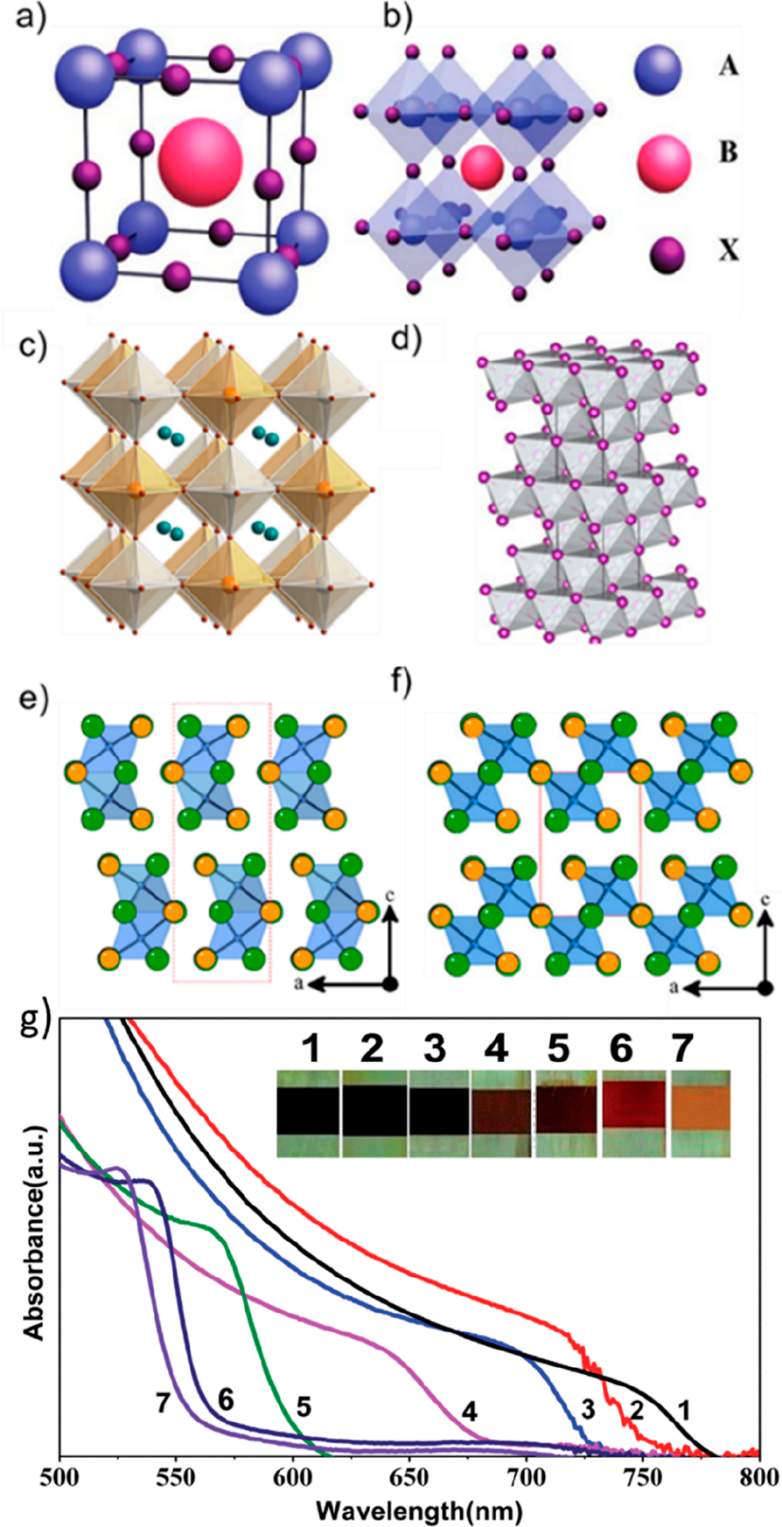
(a) Unit cell of deal cubic ABX_3_ perovskite, and (b) ABX_3_ cubic perovskite crystal structure. Reproduced with permission from ref (112). Copyright 2018 Wiley. (c) Crystal structure of A_2_B′BX_6_ double perovskites. Orange for B, gray for B′, turquoise for A, and brown for X. Reproduced with permission from ref (113). Copyright 2016 American Chemical Society. (d) Crystal structure of the A_*a*_B_*b*_X_*x*_ halide rudorffites. Reproduced with permission from ref (104). Copyright 2017 Wiley. (e) Crystal structure of 0D A_3_B_2_X_9_ nonperovskite. (f) 2D layered structure of A_3_B_2_X_9_ vacancy-ordered perovskite. Orange for A, green for X, and blue for B. Reproduced with permission from ref (109). Copyright 2015 American Chemical Society. (g) UV–vis absorption spectra of mixed halide lead perovskite (MAPb(I_1–*x*_Br_*x*_)_3_) films. The numbers 1–7 denote samples corresponding with increasing bromine content (X) within the films. Reproduced with permission from ref (81). Copyright 2014 Royal Society of Chemistry.

The first synthetic perovskite oxide, CaTiO_3_, was synthesized in 1851 using a flux growth process.^74^ Later on, the first MHPs, with composition CsPbX_3_ (X = Br, Cl, I), were synthesized from aqueous solution by Wells et al. as early as 1893,^75,76^ but their perovskite structure was confirmed in 1957 by Møller.^75^ The first organometallic halide perovskites with methylammonium as the A-site cation were synthesized by Weber and co-workers two decades later (1978).^77^ In a typical MHP, the A-site of the ABX_3_ structure (Figure 2a) can be occupied by inorganic monovalent cations (such as cesium in CsPbI_3_), organic cations (such as methylammonium or formamidinium in MAPbI_3_ and FAPbI_3_, respectively), or hybrid organic–inorganic monovalent cations (Cs_*x*_(MA_0. 17_FA_0.83_)_(1–*x*)_Pb(I_0.83_Br_0.17_)_3_, with *x* = 5%). The B site is occupied by an inorganic divalent cation, such as Pb^2+^, Sn^2+^, or Ge^2+^. Finally, the X site is occupied by a halide anion (Br^–^, Cl^–^, and I^–^) or by a combination of these anions. The cubic crystal structure of ABX_3_ is given in Figure 2b. Recently, MHPs have become extremely popular due to intensive research into their synthesis, compositional engineering, and applications. MHPs exhibit remarkable optical properties, including a high optical absorption coefficient and the ability to absorb light across a broad spectrum ranging from the visible to the near-infrared region of the electromagnetic spectrum. Additionally, the band gaps of perovskites can be easily tuned through chemical composition engineering. The defect-tolerant nature of MHPs allows for large carrier diffusion lengths ((∼100 nm – ∼1 μm) for polycrystalline film and to over 100 μm for single crystals.^11,78,79^ MHPs typically exhibit low exciton binding energies, which means that low energy is required for exciton dissociation into free charge carriers (electrons and holes), indicative of efficient charge separation.^80^ Band gap tunability can be achieved through partial or full substitution at the halide site or A-site cation. For instance, replacing iodide with bromide in MAPbI_3_ may lead to a blue shift of band edge and increase the band gap (Figure 2g),^81^ while substituting MA with FA may lead to a red shift and decrease of the band edge.^82^ This band gap tunability along with other outstanding optical properties of MHPs enable the control of perovskite properties for various optoelectronic applications.

The structural dimensionality of a material is related to the atoms’ arrangement and connections, which affect the crystal structure, bonding types, and defects of the material. MHPs can exist in various structural dimensionalities, including 3D, 2D, 1D, and 0D. In contrast, the electronic dimensionality describes the connection between the atomic orbitals comprising the lower conduction band (LCB) and upper valence band (UVB), which impact the material’s band gap and carrier transport.^9,83^ Due to the isolation of the octahedra, in materials with 0D electronic dimensions, the LCB and UVB are nondispersive in all directions and the electrons are confined within all three spatial dimensions, producing distinct energy levels and quantum phenomena. Cs_4_PbBr_6_, exhibiting a 0D both in its electronic and structural dimensions, comprises isolated [PbBr_6_]^4–^ octahedra isolated by Cs^+^ ions. In comparison with its 3D counterpart, 0D Cs_4_PbBr_6_ exhibits a hundred times higher photoluminescence quantum yield^84^ due to large exciton binding energy.^85^ In the case of MHPs with 1D electronic and structure dimensions, the CBM and VBM overlap only along the chains of octahedra but not perpendicularly, and the electron motion is thus confined to one direction. Consequently, the LCB and UVB are narrower, resulting in a larger band gap than that of their 3D and 2D counterparts.^9,86^ In 2D MHPs, the organic cations act as insulating spacers, and electrons are allowed to move within two dimensions and are confined in the third dimension. In comparison to 3D perovskites, the presence of organic spacers in 2D perovskites enhances the ambient stability and affects the structural distortion that can control the band edge states.^87,88^

Although many materials that possess 3D structures may be electronically 3D, having a 3D structure does not guarantee electronic three-dimensionality and vice versa. The mismatch between the structural and electronic dimensions is clearly visible in the lead-free double halide perovskite Cs_2_AgBiBr_6_ (Figure 2c). This material, despite its 3D structure, is electronically low dimensional (LD) and has an indirect band gap.^89,90^ The large intermediate band gap and low electronic dimensionality of Cs_2_AgBiX_6_ were found to be due to the orbital mismatch between Ag and Bi.^15,91^ The transition from an indirect to a direct band gap for Cs_2_AgBiBr_6_ can be achieved by lowering the structural dimensionality with specific organic spacer cations, such as butylammonium, allowing for a layered 2D crystal structure.^14^ However, LD structures also have low electronic dimensionality, leading to large band gaps and large effective carrier masses.^89,90^

Notwithstanding the excellent intrinsic properties of lead halide perovskites (LHPs) that make promising semiconductors, the toxicity and instability are by far the biggest challenges to be urgently addressed.^92,93^ Researchers have explored low-toxicity alternatives, such as divalent metal cations like germanium (Ge^2+^) and tin (Sn^2+^), to replace Pb^2+^ and retain the perovskite crystal structure of ABX_3_. These lead-free materials are called 3D perovskite-like materials.^17,94^ Sn^2+^-based perovskites, such as MASnI_3_, CsSnI_3_, have direct band gaps between 1.2 and 1.4 eV.^94−96^ On another hand, Ge^2+^-based structures, like CsGeI_3_, MAGeI_3_, and FAGeI_3_, show direct band gaps between 1.6 and 2.4 eV.^97^ However, both Ge and Sn suffer from rapid oxidation when exposed to the ambient air.^17,97^ Since Sn^2+^/Sn^4+^ has a low redox potential (−0.15 V), Sn^2+^ is easily oxidized to Sn^4+^ when exposed to air, and this process is accelerated by the presence of H_2_O. The presence of Sn^4+^ leads to the destruction of the perovskite structure, which adversely affects the stability of the material.^98,99^ Like Sn^2+^, Ge^2+^ is not stable and tends to oxidize to the stable Ge^4+^ following the same trend as the Sn^2+^-based perovskite. Therefore, finding low-toxicity and stable absorption materials is of great interest. The replacement of the cations in the A-site by larger organic cations, e.g., butylammonium (BA) and 2-phenyl-ethylammonium (PEA), in Ge^2+^- and Sn^2+^-based perovskite results in a low dimensionality structure and a widening of the direct band gap to (>2). (PEA)_2_SnI_4_ and (PEA)_2_GeI_4_ exhibit 2D perovskite structures and have high stability to oxidation compared to 3D counterpart perovskites.^100,101^

Antimony (Sb) and bismuth (Bi) are of great interest as lead-free perovskite-inspired materials (PIMs) because both Bi^3+^ and Sb^3+^ have electron structures similar to those of Pb^2+^ and are less toxic than lead, more stable, and abundant enough for large-scale use. Their electronic structure is responsible for the interesting optoelectronic properties. PIMs may not have the same crystal structure and/or ABX_3_ composition, like LHPs. Indeed, the replacement of lead by Bi^3+^ or Sb^3+^ results in a low-dimensional structure, differing from the three-dimensional structure of ABX_3_ perovskites.^102,103^

Among the various perovskite-inspired materials, iodobismuthates, 2D layered structures, and nonperovskite 0D structures have received widespread attention as potential lead-based alternatives, especially for photovoltaic applications. Iodobismuthates, often crystallizing in the NaVO_2_ rudorffite structure, are a class of PIMs based on the ternary Ag–Bi–I system with the general formula of Ag_*a*_Bi_*b*_X_*a*+3*b*_. Rudorffites (Figure 2d) have 3D structures based on edge-shared octahedra [AX_6_] and [BX_6_] octahedra and have direct band gaps of <2 eV.^104^ Several rudorffite materials have been investigated for photovoltaic application, such as AgBi_2_I_7_,^105^ Ag_3_BiI_6_,^104^ AgBiI_4_,^106,107^ and Ag_2_BiI_5_,^106,108^

Bi^3+^ or Sb^3+^ compounds with the A_3_B_2_X_9_ chemical composition crystallize in 2D perovskite or 0D nonperovskite polymorphs, depending on the preparation method. The 2D layered structure of A_3_B_2_(V)X_9_ is also known as vacancy-ordered perovskite, in which the ratio of B-site cations to vacancies is 2:1 while the 0D structure consists of pairs of isolated face-sharing [B_2_I_9_]^3–^octahedra is surrounded by A.^108^ Starting from the most common example of A_3_B_2_X_9,_ Cs_3_Sb_2_I_9_ exhibited both 2D layered perovskite (Figure 2f) and 0D nonperovskite structure (Figure 2e); while the layered 2D perovskite structure is formed by vapor deposition, the 0D nonperovskite structure is easily produced via solution processing.^109^ 0D Cs_3_Sb_2_I_9_ is thermodynamically more favorable in synthesis than 2D, but 2D Cs_3_Sb_2_I_9_ shows greater potential for optoelectronic properties than 0D.^109,110^ The 0D/2D Cs_3_Sb_2_I_9_, 2D MA_3_Sb_2_I_9_, 0D Cs_3_Bi_2_I_9_, and 0D MA_3_Bi_2_I_9_ are the most studied compounds of the A_3_B_2_I_9_ class of materials.^109,111^

Synthesizing polycrystalline MHPs can be achieved through either solution deposition or vacuum deposition methods. Solution deposition involves using various techniques such as spin-coating, drop-casting, hydrothermal synthesis, solvothermal synthesis, ultrasound-assisted synthesis, and microwave-assisted synthesis to create thin films from precursor solutions. On the other hand, vacuum deposition methods involve synthesizing MHPs through coevaporation under high vacuum conditions. Vacuum deposition methods can be further categorized into thermal evaporation, pulsed laser deposition, and chemical vapor deposition, whereas the colloidal nanocrystal can be synthesized by hot-injection and ligand-assisted reprecipitation.^114−116^

### 3.2 Halide Perovskite for Water Splitting

Over the past decade, halide perovskites led to unprecedented advances in a variety of optoelectronic applications, such as solar cells,^117^ light-emitting diodes (LEDs),^118^ photodetectors,^119^ and PEC water splitting with an increasing interest.^120^ Similar to conventional PEC cells using metal oxides (e.g., TiO_2_ or WO_3_) as the photoelectrode, a typical halide perovskite PEC cell consists of a working electrode (WE) (i.e., a halide perovskite-based photoelectrode), a counter electrode (CE), and a reference electrode (RE). This type of photoelectrode structure effectively solves the critical stability problems of halide perovskites immersed in aqueous electrolytes by easily covering a waterproof and conductive protective layer on top of the halide perovskite layer. To date, many efforts have been made to stabilize halide perovskite photoelectrodes by depositing different types of such protective layers, e.g., Ni thin films (∼8 nm), field metal (FM),^121^ Ti foil,^122^ graphite epoxy^123,124^ or pyrolytic graphite,^125^ and carbon-based coating layers^126−128^ on halide perovskite photoanodes in the n-i-p structure or halide perovskite photocathodes in the p-i-n counterpart.

In the following section, we briefly introduced the fabrication of PEC cells based on halide perovskites in both thin films and nanocrystals, which are classified in terms of target reactions, i.e., the evolution of oxygen and the evolution of hydrogen, respectively.

#### 3.2.1 Halide Perovskite-Based Photoanodes for Oxygen Evolution

To reach oxygen evolution by photoelectrochemical oxidation of water, PEC cells with halide perovskites in n-and-p configuration are used as photoanodes. Typically, photogenerated holes in the perovskite layer are transported to the corresponding electrode via hole-transport layers (HTLs), which oxidize oxygen ions supplied by the aqueous electrolyte. As described above, the high susceptibility of halide perovskites to water is considered as one of the major barriers for their use in PEC cells. To date, significant research has been conducted to protect halide perovskite-based photoelectrodes within PEC cells by coating them with a water-resistant and ideally electrocatalytic layer. In 2015, Da and co-workers^129^ assembled, for the first time, a CH_3_NH_3_PbI_3_ (MAPbI_3_)-based multilayer photoanode coated with an ultrathin Ni layer (∼8 nm), which serves as both a waterproof shield and Ohmic contact.

**Figure 3:**
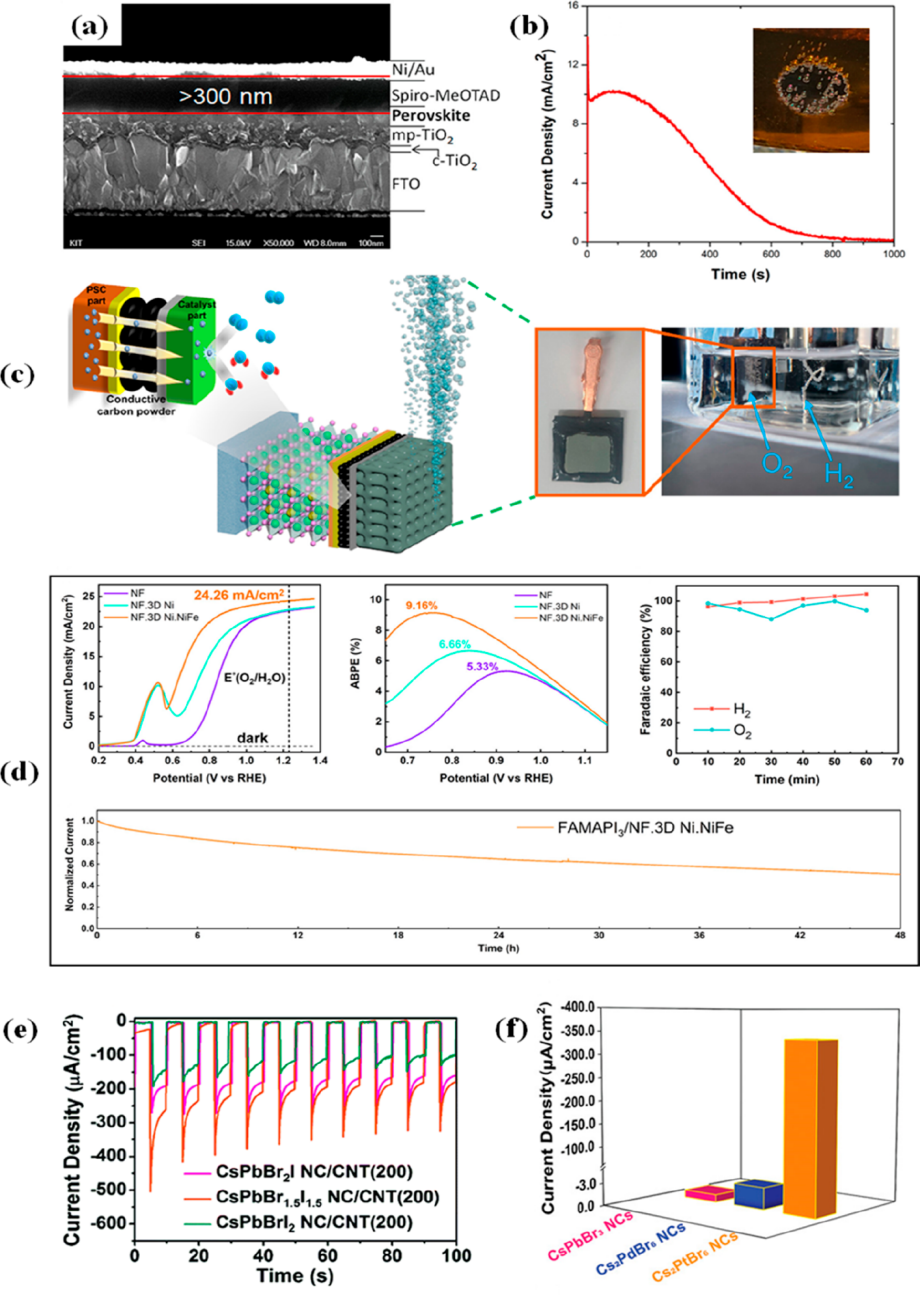
(a) SEM cross-sectional image of the MAPbI_3_-based photoanode. (b) Photocurrent density vs time graph of perovskite photoanode under illumination (0.7 sun) and bias (1.0 V vs SHE). Reproduced with permission from ref (131). Copyright 2016 American Chemical Society. (c) Schematic illustration (left) and photo (right) of the perovskite photoanode configuration for PEC O_2_ evolution.^134^ (d) Top left: Photocurrent density; top middle: applied bias photon-to-current conversion efficiency (ABPE), and top right: faradaic efficiency of the FAMAPbI_3_-based photoanode. Bottom: Photocurrent density vs time graph under 1 sun illumination at 1.23 V_RHE_ of the FAMAPI_3_-based photoanode. Reproduced with permission from ref (134). Copyright 2022 American Chemical Society. (e) Amperometric *I–t* curves of the CsPbBr_*x*_I_3–*x*_ NC/carbon nanotube at −0.4 V. Reproduced with permission from ref (135). Copyright 2019 Royal Society of Chemistry. (f) Current densities of different halide perovskite nanocrystal-based PEC systems. Reproduced with permission from ref (137). Copyright 2021 Wiley-VCH.

A record photocurrent density of >10 mA cm^–2^ was achieved in 0.1 M Na_2_S at 0 V_Ag/AgCl_ under 1 sun illumination (100 mW cm^–2^), and it retained >2 mA cm^–2^ after 15–20 min of illumination using bias. This finding indicated that the Ni thin film could be a promising candidate as a protective catalytic layer for halide perovskite photoelectrodes to be used in water splitting reactions. Wang and co-workers^130^ further improved the stability of the MAPbI_3_-based PEC system using both Ni-coating and functionalization of the perovskite surface with hydrophobic alkylammonium cations (tetraethylammonium (TEA) and tetrabutylammonium (TBA)). The TEA-treated perovskite photoanodes can sustain PEC water oxidation for about 30 min but with an unexpectedly low photocurrent density of 2 mA cm^–2^, which is probably attributed to a decrease in the conductivity of the protective layers. On the other hand, many holes were created on the surface of the perovskite after TBA treatment, resulting in incomplete Ni coverage and thus a decrease in PEC efficiency (photocurrent of only 0.42 mA cm^–2^); it is important to achieve a morphology without holes on top of the perovskite layer, which can effectively impede water penetration. Hoang and co-workers^131^ fabricated a MAPbI_3_-based photoelectrode with a thick (>300 nm, see cross-sectional SEM image in Figure 3a) and dense spiro-OMeTAD HTL, which greatly improves the stability of the perovskite photoanode with a long lifetime of almost 60 min (Figure 3b) while maintaining the water oxidation reaction.

Later in 2018, the same group^132^ used a mold-cast and life-off process to introduce a low-melting-point Field’s metal (FM) layer sandwiched by a perovskite layer and Ni shielding, which strikingly stabilizes the perovskite photoanode in a harsh oxidative environment for more than 10 h. As the FM lacks catalyst activity for oxygen evolution, the authors optimized a thickness of ∼10 μm for the catalytic Ni layer, achieving a constant photocurrent of 13 mA cm^–2^ for about 6 h under illumination (0.7 sun), as well as a bias of 1.3 V_RHE_ (RHE = reversible hydrogen electrode) in a KOH electrolyte.

As an alternative to metal-based shielding, low-cost carbon-based materials have also been used as excellent protective layers as well as electrocatalytic layers by insulating the perovskite from the aqueous solution. For instance, Tao and coauthors^133^ used a mixed cationic perovskite (5-AVA)_*x*_(MA)_1–*x*_PbI_3_ [5-AVA = HOOC(CH_2_)_4_NH_3_^+^]^−^-based photoanode encapsulated with conductive carbon paste and silver coating for both as water-resistant and hole transporting medium; they achieved a high photocurrent density of 12.4 mA cm^–2^ at 1.23 V_RHE_ in a KOH solution under 1 sun illumination, while demonstrating the unprecedented stability that PEC performance can be continuously maintained with a steady-state response for >12 h. It is well-known that the implementation of water splitting should meet the operational requirement of 1.23 V. Therefore, halogen perovskites with a wide bandwidth, e.g., CsPbBr_3_, have recently attracted more attention for application in PEC cells. Poli and co-workers constructed a TiO_2_|CsPbBr_3_|mesoporous carbon (m-c)|graphite plate (GS) photoanode, demonstrating a constant photocurrent of over 2 mA cm^–2^ at 1.23 V_RHE_ under continuous illumination (AM 1.5 G) with an outstanding stability of >30 h in a liquid electrolyte. More interestingly, the authors modified the GS with an Ir-based water oxidation catalyst (WOC), which led to a decrease in *E*_onset_ (initial potential) of about 100 mV with increased photovoltage (>1.3 V). More recently, Kim and co-workers^134^ used conductive carbon powder (CCP) as a coupler between a monolithic photodiode based on double cationic perovskite (FA_0.93_MA_0.07_PbI_3_) and a robust NiFe-layered Ni film catalyst (Figure 3c). The fabricated perovskite photoelectrode shows an *E*_onset_ of 0.56 V and an extremely high photocurrent density of >24 mA cm^–2^ at 1.23 V_RHE_, with a promising photocurrent efficiency (ABPE) of 9.16% (see Figure 3d). This work also highlights an extremely stable lifetime of 48 h for the PEC performance under constant illumination.

Compared to the widespread use of bulk 3D halide perovskite layers in PEC cells, the use of nanocrystalline (NC) perovskite-based layers as photoelectrodes is still in its infancy due to the difficulty of fabricating high-quality NCs perovskite layers as well as high water susceptibility. In contrast, their suspension-phase counterparts have been extensively studied for conventional photocatalytic reactions. Yang and co-workers^135^ investigated the photoelectrochemical activity of hybrid photoelectrodes based on CsPbBrxI_3–*x*_ NCs/carbon nanotubes (CNTs), obtaining a reasonable photocurrent density of 417 μA cm^–2^, which shows an almost 8-fold increase compared to the initial CsPbBr_3_ NC composite without CNTs at −0.4 V_Ag/AgCl_ under calibrated illumination of 150 mW cm^–2^ (see Figure 3e). Furthermore, instead of coating protective surface layers, the development of stable halide perovskites alone is still strongly requested to create sustainable PEC cells.

Lead-free halide perovskites in both bulk and nanocrystal forms have shown their great potential in fulfilling this intrinsic requirement. Hamdan and co-workers^136^ prepared a thin photodiode based on a 3D Cs_2_PtI_6_ perovskite thin film without any protective layers for water oxidation, showing extremely high stability against high temperature and extremely acidic and basic electrolytes (pH = 11). A moderate photocurrent density of 0.8 mA cm^–2^ was obtained at 1.23 V_RHE_ (100 mW cm^–2^) with continuous operation for more than 12 h. In addition, Peng and co-workers^137^ synthesized ligand-free Cs_2_PtBr_6_ NCs, with a corresponding band gap of 1.32 eV with excellent conductivity, which are highly stable against high temperature, moisture and light radiation; the photoelectrodes based on Cs_2_PtBr_6_ NCs in PEC tests achieved a maximum photocurrent density of 335 μA cm^–2^ at −0.6 V_Ag/AgCl_ under LED light (10.18 mW cm^–2^), dramatically outperforming PEC systems based on CsPbBr_3_ NCs and Cs_2_PdBr_6_ NCs (see comparison of photocurrent densities in Figure 3f). More recently, lead-free double perovskite nanocrystals, i.e., Cs_4_CuSb_2_C1_12_ NCs,^138^ also showed promising photoelectrochemical performance, due to the nature of the direct band gap together with the lower effective mass, which was realized by transforming bulk Cs_4_CuSb_2_C_12_ crystals into nanocrystals. We summarize the halide perovskite-based photoanodes in terms of water oxidation performance in Table 1. As it can be noticed, both photoanode- and photocathode-based halide perovskites can reach and even overcome the value of 10 mA/cm^2^, which has been assumed as a break-even point for a given investment.^139^

**Table 1:** Summary of Representative Water Splitting Studies on Halide Perovskite-Based PEC Cells

Reaction type	PEC cell configuration	Electrolyte	Irradiation	Onset potential	Photocurrent density	Faradic efficiency	Duration	Ref.
Water oxidation	FTO|TiO_2_|MAPbI_3_|Spiro-OMeTAD|Au|Ni	0.1 M Na_2_S	100 mW cm^–2^ (AM 1.5G)	N/A	>10 mA cm^–2^ @ 0 V_Ag/AgCl_	N/A	0.25–0.33 h	(129)
	FTO|TEA-modified MAPbI_3_|Spiro-OMeTAD|Ag|Ni	0.1 M Na_2_S	100 mW cm^–2^ (AM 1.5G)	N/A	2.1 mA cm^–2^ @ 0 V_Ag/AgCl_	N/A	>0.5 h	(130)
	FTO|c-TiO_2_|m-TiO_2_|MAPbI_3_|Spiro-OMeTAD|Au|Ni	K-Borate/1.0 M KOH	0.7 sun illumination	0.5 V_SHE_	17 mA cm^–2^@ 1.23 V_SHE_	N/A	1 h	(131)
	FTO|TiO_2_|MAPbI_3_|Spiro-OMeTAD|Au|FM|Ni	1.0 M KOH	0.7 sun illumination	0.75 V_RHE_	13 mA cm^–2^ @ 1.3 V_RHE_	N/A	6 h	(132)
	FTO|TiO_2_|(5-AVA)_*x*_(MA)_1–*x*_PbI_3_|conductive carbon|sliver paint|carbon	1.0 M KOH	100 mW cm^–2^ (AM 1.5G)	0.8 V_RHE_	12.4 mA cm^–2^ @ 1.23 V_RHE_	82%	>48 h	(133)
	FTO|TiO_2_|CsPbBr_3_|m-carbon|graphite sheet|Ir-based WOC	0.1 M KNO_3_	100 mW cm^–2^ (AM 1.5G)	0.6 V_RHE_	2 mA cm^–2^ @ 1.23 V_RHE_	N/A	30 h	(140)
	FTO|SnO_2_|FA_0.93_MA_0.07_PbI_3_|Spiro-OMeTAD|Au|conductive carbon powder|Ni foil (NiFe)	1 M KOH (pH = 14)	100 mW cm^–2^ (AM 1.5G)	1.5 V_RHE_	24.26 mA cm^–2^ @ 1.23 V_RHE_	∼100%	48 h	(134)
	FTO|CsPbBr_*x*_I_3–*x*_ NC/carbon nanotube	0.1 M TBAPF_6_	150 mW cm^–2^ (AM 1.5G)	N/A	0.417 mA cm^–2^ @ −0.4 V_Ag/AgCl_	N/A	>0.03 h	(135)
	FTO|TiO_2_|Cs_2_PtI_6_	1 M KOH (pH = 11)	AM 1.5G	1.1 V_Ag/AgCl_	0.8 mA cm^–2^ @ 1.23 V_RHE_	N/A	>12 h	(136)
	Cs_2_PtBr_6_ NC|glassy carbon electrode	0.1 M phosphate buffer solution	10.18 mW cm^–2^ (365–370 nm LED light)	0.5 V_Ag/AgCl_	0.335 mA cm^–2^ @ −0.6 V_Ag/AgCl_	N/A	>0.16 h	(137)
	FTO|Cs_4_CuSb_2_Cl_12_ NC	0.1 M NH_4_PF_6_	100 mW cm^–2^ (AM 1.5G)	N/A	0.006 mA cm^–2^ @ −0.85 V_Ag/AgCl_	N/A	>0.16 h	(138)
	Pt|graphite epoxy|CsFAMA|FTO|glass|FTO	0.1 M KBi, 0.1 M K2SO4 buffer (pH = 8.5)	100 mW cm^–2^ (AM 1.5G)	1.8 V_RHE_	15 mA cm^–2^@ 1.23 V_RHE_	N/A	96 h	(123)
Water reduction	FTO|PEDOT:PSS|MAPbI_3_|PCBM|Ag|FM|Pt	0.1 M borate	100 mW cm^–2^ (AM 1.5G), λ > 400 nm	0.7 V_RHE_	9.8 mA cm^–2^ @ 0 V_RHE_	95.1%	1.8 h	(121)
	FTO|NiO_*x*_|CsFAMA perovskite|PCBM|FM|Pt-BiVO_4_|TiCo	0.1 M borate, K_2_SO_4_	100 mW cm^–2^ (AM 1.5G)	–0.6 V	0.39 mA cm^–2^ @ no bias	78.8% Solar-to-hydrogen (STH) efficiency = 0.35%	18 h	(141)
	FTO|NiO|CsPbBr_3_|ZnO|Ag|FM|Pt	0.2 M Na_2_HPO_4_/NaH_2_PO_4_	100 mW cm^–2^ (AM 1.5G)	1.16 V_RHE_	1.2 mA cm^–2^ @ 0 V_RHE_	90%	>1 h	(122)
	ITO|NiO|MAPbI_3_|PCBM|Ag|silver paste|Ti foil|Pt	0.5 M H_2_SO_4_	100 mW cm^–2^ (AM 1.5G)	0.95 V_RHE_	18 mA cm^–2^ @ 0 V_RHE_	∼100%	12 h	(142)
	FTO|Cs_2_SnI_6_	0.3 M NaCl	150 mW cm^–2^ (AM 1.5G)	–0.15 V_Hg/Hg2Cl2_	0.92 mA cm^–2^ @ −0.8 V_Hg/Hg2Cl2_	Energy conversion efficiency = 0.54%	N/A	(143)
	APTPE/IrOx (tandem junction)	0.5 M H_2_SO_4_	100 mW cm^–2^ (AM 1.5G)	2.1 V_RHE_	12.5 mA cm^–2^ @ 0 V_RHE_	STH efficiency = 15%	120 h	(128)

#### 3.2.2 Halide Perovskite-Based Photocathodes for Hydrogen Evolution

**Figure 4:**
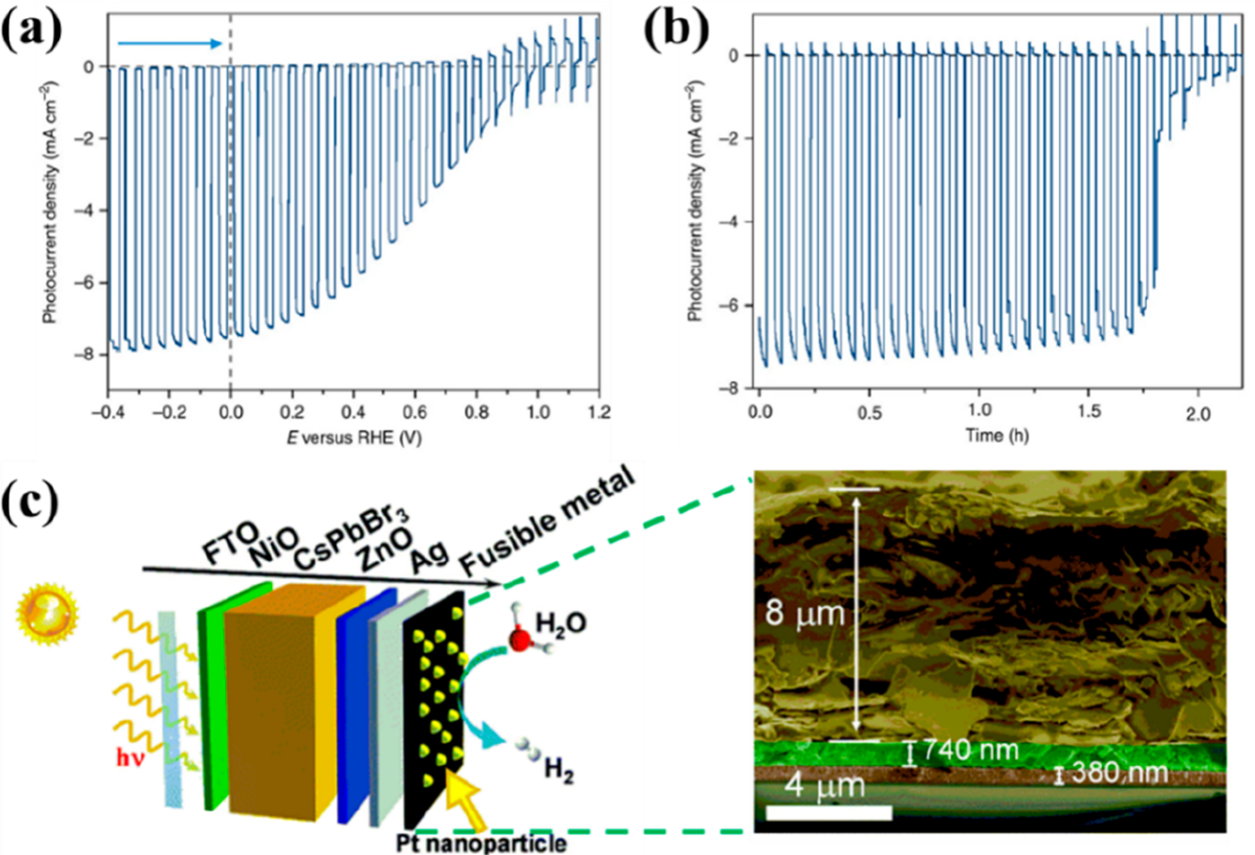
(a) Photocurrent density vs applied bias graph of the MAPbI_3_-based photocathode. (b) Photocurrent density vs time graph recorded at 0 V_RHE_. Reproduced with permission from ref (121). Copyright 2016 Nature. (c) Schematic structure and the cross-sectional SEM image of the CsPbBr_3_-based photocathode. Reproduced with permission from ref (141). Copyright 2018 Royal Society of Chemistry.

In a PEC system based on a halide perovskite, the p-i-n configuration aims to reduce water through the inverse principle of the n-i-p structure, which transports photogenerated electrons from the perovskite layer to the surface electrode via an ETL such as a PCBM or C_60_. Similar to the photoanode, FM is widely used as an effective protective layer to protect the perovskite photocathode. As the first example, Quesada and co-workers^121^ placed Pt nanoparticles as electrocatalysts on a MAPbI_3_-based photocathode, achieving a relatively high current density of 9.8 mA cm^–2^ at 0 V_RHE_ and *E*_onset_ of 0.95 V (Figure 4a,b). The Pt-coated perovskite photocathode showed high stability with 80% retention of its initial photocurrent for about 1 h under constant simulated sunlight (AM = 1.5 G). Later in 2018, the same group^144^ used a state-of-the-art photocathode based on a triple cation halide perovskite (CsFAMA) with a BiVO_4_|TiCo photoanode to fabricate a tandem PEC system. The prepared tandem PEC cell with a large area of 10 cm^2^ was able to operate continuously for almost 20 h with an STH efficiency of 0.35% and a small decrease in photocurrent density. Gao and co-workers^141^ used Pt nanoparticles as FM to fabricate a fully inorganic photocathode based on CsPbBr_3_ perovskite, using metal oxides as charge transport layers, i.e., NiO for the HTL and ZnO for the ETL (see PEC cell structure in Figure 4c). The fully inorganic perovskite photocathode achieved a photocurrent density of 1.2 mA cm^–2^ at 0 V_RHE_, and it can maintain ∼94% activity after 1 h of continuous illumination.

In addition to the heavily used FM, a low-cost Ti film has been recognized as another type of attractive protective material with high conductivity and photochemical stability. Zhang and co-workers^122^ demonstrated a scalable method to fabricate a sandwich-type photocathode based on MAPbI_3_ coated with Ti film, exhibiting an *E*_onset_ of 0.95 V_RHE_ with an excellent photocurrent density of 18 mA cm^–2^ at 0 V_RHE_ in an H_2_SO_4_-based electrolyte under irradiation of 1 sun. The Ti film-protected photocathode showed high stability after 12 h of continuous light irradiation in aqueous solution (pH: 7–13.6). Regarding the use of lead-free halide perovskites in the production of photocathodes, Dang and co-workers^142^ synthesized a Cs_2_SnI_6_ (Sn^4+^) thin film with a high thermal stable phase instead of the widely known yet highly unstable CsSnI_3_ (Sn^2+^)^143^ under ambient conditions. The Cs_2_SnI_6_ photocathode achieved a moderate photocurrent density of 0.92 mA cm^–2^ and an energy conversion efficiency of 0.54% at −0.8 V Hg/Hg_2_C_12_ in a NaCl solution, which was attributed to the dense domain of the active sites as well as the slow relaxation dynamics in the excited state through the in-band channel. In order to show a correlation between structure and electronic properties of perovskite photoanodes and halide perovskite-based photocathodes, a comparison of their performance in terms of hydrogen evolution efficiency is also summarized in Table 1.

### 3.3 Halide Perovskite Electrodes for CO_2_ Reduction

**Figure 5:**
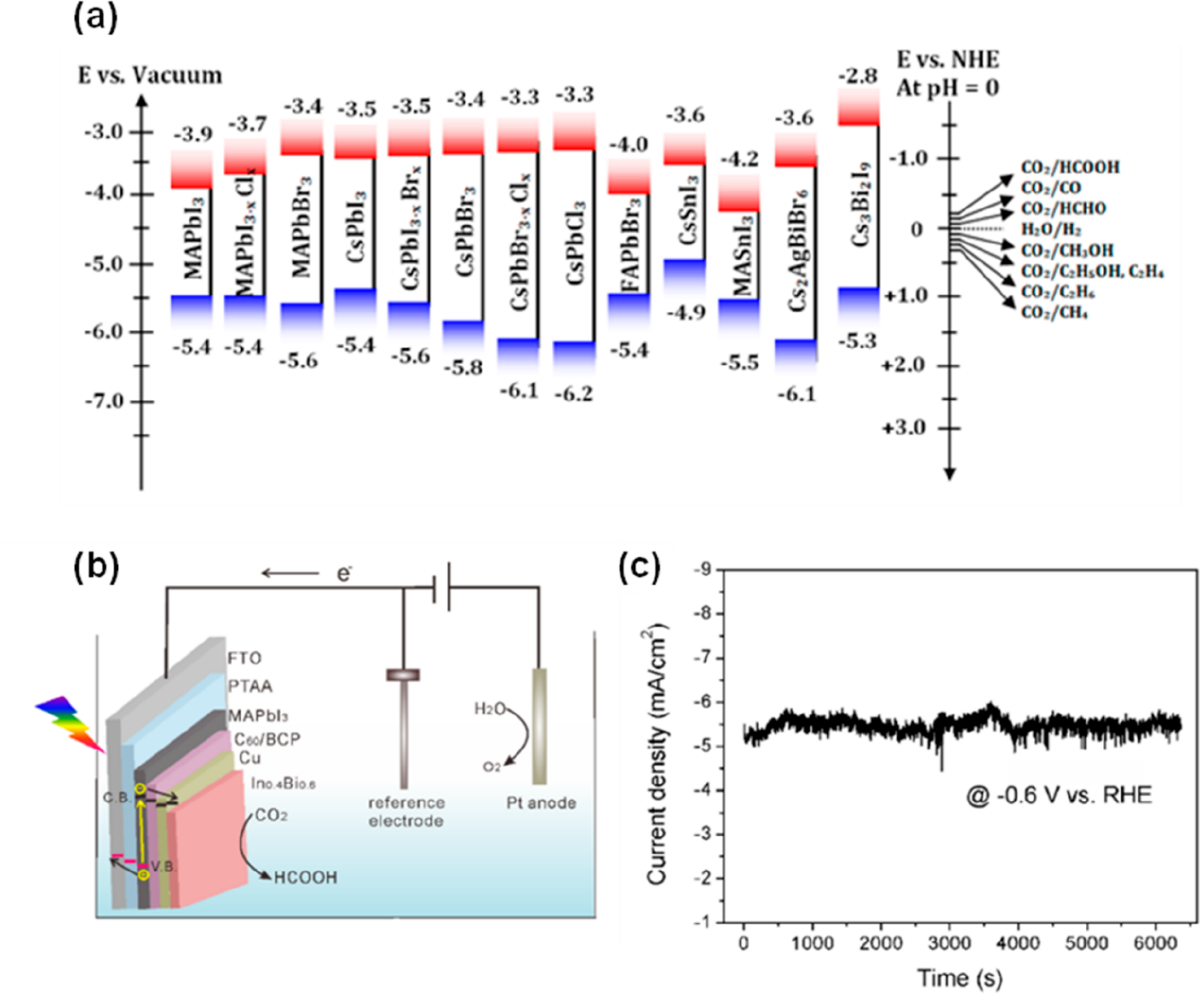
(a) CB and VB positions of various halide perovskite compositions, with respect to both vacuum and NHE levels. The CO_2_ redox levels are also present. Reproduced with permission from ref (21). Copyright 2020 American Chemical Society. (b) PEC setup of the In_0.4_Bi_0.6_-coated halide perovskite photocathode in a standard three-electrode system. The photocathode is illuminated from the FTO side. The direction of electron or hole flow in the cell is shown. (c) Stability test of the perovskite/In_0.4_Bi_0.6_ photocathode under 1 sun illumination at −0.6 V vs RHE. Reproduced with permission from ref (150). Copyright 2019 American Chemical Society.

The activation of a CO_2_ molecule into a CO_2_^**·**–^ intermediate requires an energy of −1.9 V vs NHE in a neutral pH medium^145^ The reduction of CO_2_ by most halide perovskites is a thermodynamically feasible reaction because their conduction bands (CBs) are at relatively higher positions (more negative CBs) than −1.9 V vs NHE, as shown in Figure 5a.^21^ Also, halide perovskites have band gaps higher than 1.34 eV, which is more than the Gibbs free energy (259 kJ mol^–1^) of the reduction reaction of CO_2_ to CO.^22^ However, a strong negative potential (−1.9 V) is necessary to overcome the energy barrier for the conversion of linear molecule O=C=O into CO_2_^**·**–^, which is the rate-determining step for the reactions. Then the CO_2_^**·**–^intermediate reacts with protons in aqueous media to form the desired product.^146^ The photocatalytic reduction of CO_2_ in aqueous media results in the formation of various products such as formic acid, formaldehyde, CO, methane (Table 2) and other C_2_–C_3_ hydrocarbons. Photoreduction of CO_2_ by halide perovskites have been studied using both particulate catalysts in suspension or photoelectrochemical cells^23,147,148^ or deposited ; CO_2_ reduction by halide perovskites in the form of a photocathode has rarely been studied. A standard photoelectrochemical cell (PEC) consists of three electrodes; i.e., a reference electrode, a working photoelectrode, and a counter electrode are used for this purpose (Figure 5b).

**Table 2:** Electrochemical Reactions Involved in the Reduction of CO_2_ with Water^a^

Equation	Product	*E*^0^ (V)	*E*^0^ (V) vs RHE
CO_2_ + e^–^ → CO_2_^**·**–^	Carbonate anion radical	–1.90	–1.492
CO_2_ + 2H^+^ + 2e^–^ → HCOOH	Formic acid	–0.61	–0.202
CO_2_ + 2H^+^ + 2e^–^ → CO + H_2_O	Carbon monoxide	–0.53	–0.122
CO_2_ + 4H^+^ + 4e^–^ → HCHO + H_2_O	Form aldehyde	–0.48	–0.072
CO_2_ + 6H^+^ + 6e^–^ → CH_3_OH + H_2_O	Methanol	–0.38	0.028
CO_2_ + 8H^+^ + 8e^–^ → CH_4_ + 2H_2_O	Methane	–0.24	0.168

a Their corresponding reduction potential (*E*^0^) (V vs NHE at pH 7) values are also provided. In addition, the equivalent *E*^0^ (V) vs RHE values are presented.

The working photocathode in the cell structure consists of a halide perovskite layer deposited by spin-coating or other printing methods,^149^ an electron transport layer and a conductive electrocatalytic layer (Figure 4c and 5b). These three layers are deposited sequentially on a conductive substrate such as FTO. When the cell is illuminated, the photoexcited electrons of the semiconducting perovskite layer participate in the CO_2_ reduction reaction while the holes (photogenerated) are used in the oxidation reaction. The first demonstration of a halide perovskite-based photocathode for CO_2_ reduction came from Bakr et al.^150^ A photocathode made of MAPbI_3_, protected by a conductive In_0.4_Bi_0.6_ layer, (Figure 5b) selectively reduced CO_2_ to formic acid, which is a commercial chemical compound and hydrogen carrier in fuel cells. In_0.4_Bi_0.6_ was found to be the best HCOOH-selective electrocatalyst among the In–Bi–Sn three-component alloying systems investigated. In addition to lower electron transfer resistance (as found by electrochemical impedance spectroscopy) and acting as a protective layer for the MAPbI_3_ photocathode, the In_0.4_Bi_0.6_ coating layer stabilizes the HCOO* intermediate in CO_2_–HCOOH reaction more than other alloys. This leads to HCOOH production with 100% Faraday efficiency (FE) at a low applied potential of −0.52 V (vs RHE) under 1-sun illumination.^150^ The device produced stable current density of 5 mA/cm^2^ for at least 1.5 h monitored at −0.6 V (vs RHE) (Figure 5c).

Subsequently, Zhang et al. used a carbon encapsulation strategy to provide a water-resistant photocathode (Cs_0.15_FA_0.85_)Pb(I_0.9_Br_0.1_)_3_ toward efficient and stable CO_2_ reduction to CO.^151^ The low-cost carbon layer enabled efficient electron transfer (photogenerated) from the perovskite layer for efficient CO_2_ reduction. Perovskite photocathode coated with a molecular cobalt phthalocyanine catalyst layer exhibited a remarkable current density of 15.5 mA cm^–2^ under an AM illumination of 1.5G (100 mW cm^–2^), and the photocathode remains stable during the continuous reaction over 25 h, which signifies the state-of-the-art performance of halide perovskite-based photocathodes. They also demonstrated that a tandem cell obtained by combining a carbon-coated perovskite photocathode with a Si-based amorphous photocathode achieved unbiased CO_2_ reduction at a photocurrent density of ≈−3 mA cm^–2^ and an energy conversion efficiency of 3.85%. Andrei et al. also studied unbiased syngas production by a tandem cell consisting of a perovskite-based photocathode and a BiVO_4_-based photoanode;^152^ a cobalt-based porphyrin catalyst immobilized on CNTs is attached to the tip of the perovskite photocathode. At a low light intensity of 0.1 sun, the photocathode selectively reduces the CO_2_ in an aqueous environment for 23 h. The tandem device realizes the conversion of solar energy to CO with an efficiency of 0.02%, suggesting that the device can function as an independent artificial leaf in pH-neutral solutions.^152^ In another report, Andrei et al. achieved a CO_2_ to CO conversion with solar-to-fuel efficiency of 0.053% using perovskite/BiVO_4_-based PEC devices.^147^ Furthermore, photocathodes made of BiOI and BiOI–BiVO_4_ tandem devices have been successfully run for several hundred hours for syngas production by CO_2_ reduction.^148^ These design guidelines pave the way for enhancing the efficiency and durability of lead halide perovskites and their low-toxicity analogues that are sensitive to moisture, particularly in the context of solar fuel generation. Table 3 summarizes the performance of halide perovskite-based photocathodes for the CO_2_ reduction reaction.

**Table 3:** Summary of Performance of Halide Perovskite-Based PEC Cells toward CO_2_ Reduction Reaction

PEC cell configuration	Electrolyte	Irradiation	Onset potential	Photocurrent density	Faradic efficiency	Duration	Ref.
FTO|PTAA|MAPbI_3_|C60/BCP|Cu| In_0.4_Bi_0.6_	0.1 M KHCO_3_	AM 1.5G	–0.15 V_RHE_	5.5 mA cm^–2^ @–0.6 V_RHE_	∼100%	≥1.5 h	(150)
FTO|NiO|(Cs_0.15_FA_0.85_)Pb(I_0.9_Br_0.1_)_3_/CoPc/CNT-C|PCBM|BCP|Au	0.5 M KHCO_3_	100 mW cm^–2^ (AM 1.5G)	0.58 V_RHE_	15.5 mA cm^–2^ @ – 0.11 V_RHE_	88%	≥25 h	(151)
FTO|NiO_*x*_|(CsFAMA)Pb(Br-I)_3_ |PCBM|PEIE|Ag|FM|Ag epoxy| CoMTPP@CNT	0.5 M KHCO_3_	100 mW cm^–2^ (AM 1.5G)	–0.2 V_RHE_	5.61 ± 2.90 mA cm^–2^ @ 0 V_RHE_	N/A	23 h	(152)
PET|ITO|PEDOT:PSS|PTAA:F4TCNQ|(CsFAMA)Pb(Br–I)_3_|PCBM| PEIE|Ag|GE|CoMTPP@CNT	0.5 M KHCO_3_	100 mW cm^–2^ (AM 1.5G)	N/A	∼7 mA cm^–2^ @ 0 V_RHE_	N/A	35 h	(147)
ITO|NiO_*x*_|BiOI|ZnO|Cr|Ag|GE| Cu_92_In_8_	0.5 M KHCO_3_	AM 1.5G	N/A	∼4–5 mA cm^–2^ @ 0 V_RHE_	∼85%	∼24 h	(148)

## 4 Challenges and Opportunities for PEC Applications of MHPs

In this part, we will briefly discuss the major challenges first and then we summarize the opportunities for the future developments in the field; considering that a significant number of review papers were published on water splitting and CO_2_ reduction,^153−159^ we will restrict ourselves with issues associated with halide perovskites applications.

### 4.1 Major Challenges to Overcome

In addition to the common challenges faced in PEC water splitting and the CO_2_ reduction process that are summarized in Section 2 and extensively presented elsewhere,^160,161^ we should also overcome two additional challenges if the MHPs will be effectively employed in water splitting or photocatalytic CO_2_ reduction: toxicity of their key component (i.e., lead, Pb) and the inherent poor environmental stability (in particular, toward moisture) due to their soft ionic structures. As we discussed in Section 3.1 in detail, a significant amount of effort has been devoted to find an alternative material to substitute Pb; low-toxic alternatives such as divalent metal cations such as Ge^2+^ and Sn^2+^, Sb^3+^ and Bi^3+^ with the electron structures similar to Pb^2+^ have been investigated extensively. The perovskite-like structures obtained by these materials have been mostly tested in photovoltaic devices and often suffered from low efficiency and stability; the same problems may be encountered in PEC applications as well. Nevertheless, the progress is remarkable in the field in terms of both material and device optimization, particularly for Sn^2+^-based perovskites. All these efforts are usually coupled with the efforts to improve the stability of MHPs toward environmental factors, especially against water. The community is still largely focused on developing next-generation Pb-free absorbers for applications in optoelectronics, as well as photocatalysis.

However, the instability of halide perovskites is still the major challenge for their use in photocatalytic and photoelectrochemical applications, even though significant progress has been made in this direction. These materials show poor stability when exposed to air, UV light, high temperature, and water. Charge mobility and high particle diffusivity can also cause instability along the crystal structure. These materials are very sensitive to UV radiation, especially in the presence of air; photodegradation begins when oxygen interacts with the perovskite under illumination.^162^ Undesired ion migrations and phase transitions due to thermal degradation can also weaken the crystal structure;^162^ exposure to high temperature mainly affects organic cations of perovskites and induces irreversible decomposition.^163^ Among all external stress factors, water is the most important cause of instability in halide perovskites due to the hygroscopic nature of amine salts, which are organic cations of perovskites.^164^ Damage to the crystal structure of the perovskite film results in the loss of optical and electrical properties; it also causes the release of toxic Pb to the environment. Therefore, as a first step, the chemical and structural stability of halide perovskites must be improved if they are to be used in PEC applications.^120^ Indeed numerous strategies have been developed to enhance stability, such as compositional engineering, 2D/3D perovskite structures, surface passivation, protective layers, and encapsulation.^162,165^

Previous studies on perovskite solar cells indicated that perovskite with mixed cations (MA, FA and Cs) showed higher stability due to uniform grain formation.^164^ Perovskites with mixed cations have also been used as a photoelectric active layer for PEC devices with an additional protective effect (discussed below) against direct contact with water.^152,166,167^ In addition, 2D/3D perovskites have been reported as promising candidates for highly stable solar cells; the 2D part of the perovskite layer enhanced stability while the 3D part maintained sufficiently high photoconversion efficiency.^168^ However, as far as PEC cells are concerned, dimensional modifications alone have not been sufficient to cope with water diffusion, despite the improvements in the crystal structure of perovskites.^130,133^ Another approach to achieving a highly stable structure is to fabricate monocrystalline perovskites. Single-crystalline perovskites are free of grain boundaries, have a low trap density, and high carrier mobility;^169^ hence, solar cells made from perovskite monocrystals usually show excellent stability in humid air.^170^ Nevertheless, a photocathode based on perovskite monocrystals also could not resist for long time during water splitting reactions.^171^ Surface passivation has also been used to improve the optoelectronic properties as well as the stability of halide perovskites. Ionic defects, organic cations, and insufficiently coordinated halide anions, on the surface of perovskites act as trap sites and cause faster degradation.^172,173^ Supramolecular passivating agents have been introduced to control these defect;^117^ molecules with Lewis acid (electron acceptor) and Lewis base (electron donor) features can successfully attach on the perovskite surface and form halogen bonds, which help to reduce charge-trapping sites, improve the crystallinity of the perovskite, and thus increase stability.^29,172−174^ Even all these strategies are going to improve the intrinsic stability of MHPs, the use of better encapsulates like graphite-epoxy paste^148^ or other protection methods against moister may be still needed.

### 4.2 Opportunities for Improvement

Remarkable improvements in the light harvesting efficiencies and stability of halide perovskites have been achieved so far in photovoltaic research. The power conversion efficiency has increased from 1% to over 26%^175^ while stability has improved from few hours to thousand hours of operation^176^ in a bit more than a decade. This trend is likely to continue in the near future because the MHP-based photovoltaics is closer to practical applications. Clearly, most of the desired properties of MHPs and challenges that should be addressed (like light harvesting efficiency, toxicity, and instability) are the same for both photovoltaics and PEC applications; any developments in one field will be beneficial for the other. This is also evident from the fact that there is about a decade time lag between the popularity of MHPs in photovoltaics and PEC applications; some improvements to overcome the inherent weaknesses of MHPs had to be achieved before their use in PEC applications. It is very likely that some portion of the future developments will also be from photovoltaic research. Consequently, one needs to monitor the developments in photovoltaics closely while trying to improve the use of MHP-like materials in PEC applications.

Astonishing developments of computational tools and infrastructure, including efficient ML algorithms, have been combined with the increasing availability of scientific data in materials databases, data repositories, and online journals as well as other computational tools like DFT, and have created an attractive avenue for the discovery of new materials including MHPs. Various ML works on screening different materials (other than halide perovskites) for photocatalytic and PEC water splitting have already appeared in the literature in recent years^177,178^ while only a few cases were involved MHPs.^179,180^ On the other hand, a significant number of a ML work covering various aspects of MHPs in photovoltaics have already been published.^164,168,181−185^ Significant portion of the published work in this field aims to screen DFT generated data for the discovery of thermodynamically stable material with proper band gap; such an approach is usually called high-throughput computational screening. Databases such as International Crystal Structure Database (ICSD),^186^ Materials Project (MP),^187^ Open Quantum Materials Database (OQMD),^188^ Atomic-FLOW for materials discovery (AFLOW),^189^ and NOMAD^190^ together with high throughput workflow management programs like Firework,^191^ Atomate,^192^ and pymatgen^193^ have been used extensively in recent years. Stability analysis is usually performed, as a first step, directly on data taken from other sources or generated using DFT (at relatively low cost/low accuracy), even if some ML predictions have also been made. However, screening for band gap usually involves calculating the band gap for a relatively small fraction of the data using more accurate (and more expensive) DFT methods; then, this part of the data set is used to train a predictive ML model to predict the band gap of remaining perovskite materials.

**Figure 6:**
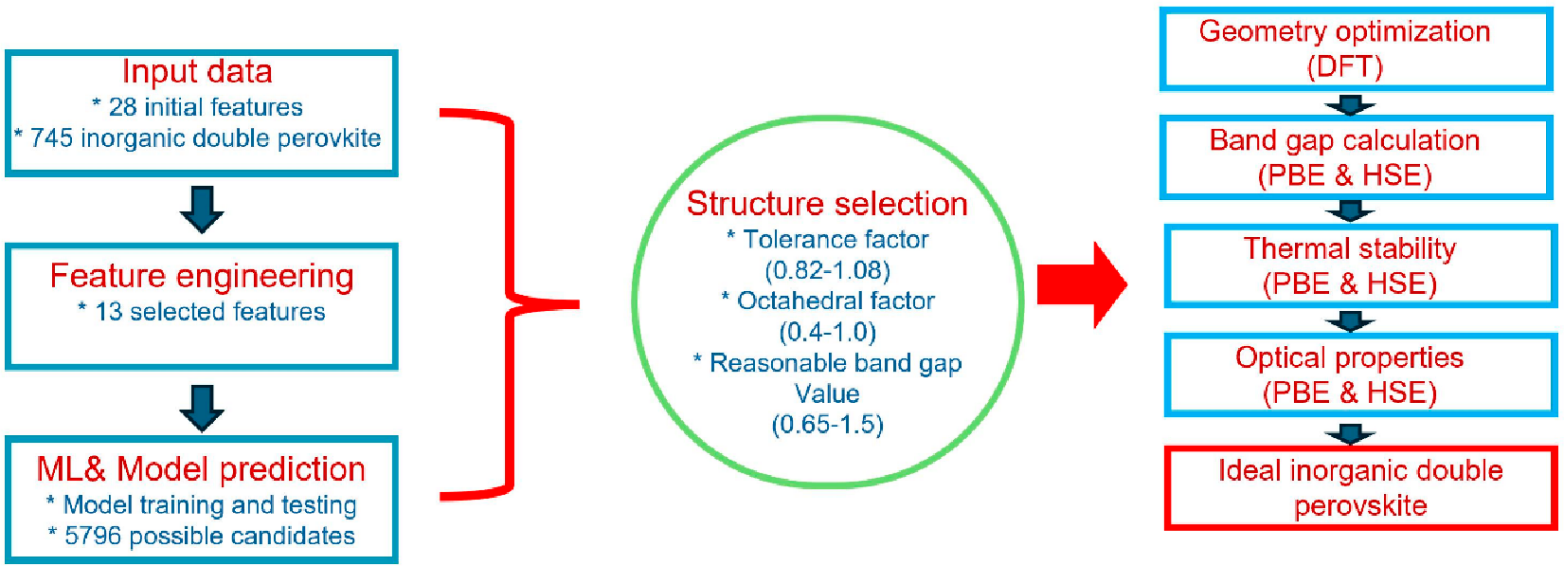
Screening flowchart employed by Gao et al. for lead-free inorganic double perovskites. Adopted from ref (196). Copyright 2021 Elsevier.

The choice of features (descriptors or input variables) is very important in the construction of an ML model; they should effectively describe the structure–property relationships. Different sets of descriptors have been used with different ML algorithms, as reported in the literature, to tackle the problem from multiple perspectives. For example, Jao et al.,^194^ used an elemental code, which is generated from a pseudopotential through a neural network autoencoder, as descriptors and constructed a boosting gradient model for band gap prediction. They used a data set of 1400 lead-free double halide perovskites, generated by high-throughput DFT simulations. Liang et al.,^195^ on the other hand, used a data set containing 469 double halide perovskites with features such as ionization potential (IP), Pauling electronegativity (EN), and atomic number (AN) to predict the stability of new perovskite structures. Similarly, Gao et al.^196^ screened 5796 double perovskites and proposed 2 candidates, Na_2_MgMnI_6_ and K_2_NaInI_6_, for use in photovoltaics; the procedure used is given in Figure 6 as an example. They used gradient boosting regression to develop a predictive model for thermodynamic stability and DFT to calculate the frequency response of 748 double halide perovskites. The descriptors were the electrochemical (such as ionization energy) and geometrical (such as ionic radius) properties of A site, B site, B′ site, and X site. The corresponding materials were also filtered according to the tolerance factor *T*_f_ between 0.82 and 1.08 and the octahedral factor *O*_f_ between 0.4 and 1.0. Compounds containing expensive and toxic materials were then eliminated. Finally, a further selection was applied according to the band gap value between 0.8 and 2.0 eV, and 2 candidates were proposed and analyzed in detail for their stability and optical and electrical properties by DFT and Ab initio molecular dynamics. Such works should also be applicable in the search for halide perovskites for photocatalytic and photoelectrochemical applications, as the performance measures are either the same (such as stability) or involve changes in only the desired value (such as band gap); this is also evident from the increasing number of publications of similar works for photocatalysis. For example, Wang et al.^197^ recently used a similar approach to find the most suitable configurations of lead free A_3_B_2_X_9_ structures for photocatalytic applications.

High-throughput experimentation (HTE), frequently coupled with ML, is another strategy that may be employed to discover or design new MHPs for photovoltaic applications. This approach is quite similar to the computational procedure described above (as their names are also suggested); it can be employed by itself or after the search space is narrowed by computational screening. Although experimental work is generally costlier and more difficult than computational methods, it is almost always needed as the final step because there is no guarantee that computationally discovered material will work in the physical world. For instance, Burger et al.^198^ argued that the materials used as batteries, biomaterials, and catalysts are mixtures of molecular and mesoscale components. Such multilength-scale complexity cannot be fully understood by molecular simulations. They used a mobile robot, driven by a Bayesian search algorithm, to improve photocatalysts for hydrogen production from water. The robot carried out 688 experiments with ten variables over 8 days, and they identified materials that had six times higher activity than the original formulations. Reviews and perspectives have been also published in recent years covering high throughput experimentation for MHPs in general^199^ or specific to photocatalytic applications.^200^ While the use of ML to analyze the data and refine the next step or batches of experiments in HTE is highly beneficial, the use of automation and robotics in synthesis and characterization is also essential as discussed by Sokol and Andrei, who also assess the common fabrication and characterization techniques used in the field by using some automation criteria.^201^

Such diversity in descriptors, tools, and approaches created a rich experience in the field that can be easily extended into new directions including PEC research. Consequently, we can expect that similar paths will be followed for PEC applications, and related works will appear in the literature with increasing numbers in the near future. As the result of similarities in the desired properties of MHPs in photovoltaics and PEC applications, some of the works already performed for photovoltaics may also be used directly in PEC processes. For instance, the toxicity and stability screening of MHPs, as frequently done for photovoltaics, will be also valid for the PEC applications, while the band gap models will need to be retrained.

## 5 Conclusions

Metal halide perovskites, hailed by many as the miracle semiconductor for photovoltaics^202^ of the past decade, exhibit outstanding optoelectronic properties (e.g., broad absorption covering the entire visible range, a tunable band gap, excellent transport properties) and easy solution chemistry from cheap and abundant precursors. They have recently been identified as ideal solid-state photocatalysts for CO_2_ reduction, but most reports refer to photocatalytic molecular systems.^24^ Only a few perovskite-based examples of photoelectrode thin films and photovoltaic-electrocatalytic systems are known. This deficiency is largely due to the well-known challenges associated with perovskites, such as the toxicity of their key component (i.e., lead, Pb) and the inherent poor environmental stability (and in particular toward moisture) due to their soft ionic structures.

Although the stability of halide perovskites remains one of the major challenges for their widespread use in photocatalytic/photoelectrochemical applications, significant progress has been made in this direction. As discussed above, various approaches have been used, such as the use of more stable components (especially cations) and structures (for example, monocrystalline structure), surface passivation, or encapsulations. We can expect more progress in this field in the near future, because halide perovskites are one of the most extensively investigated materials in recent years thanks to their enormous potential in solar cell applications. The research focus on these materials significantly shifted to stability in recent years after the efficiency improved to an acceptable level, making the stability to be the major bottleneck; this creates a big opportunity for the PEC applications of halide perovskites as well because no material could have attracted that much attention and resources if it was related to only PEC applications.

Experimental works combined with ML/DFT analysis can be the most beneficial approach for the effective use of halide perovskites in PEC applications. As we briefly reviewed, a significant amount of research has been carried out to develop effective strategies for the stability of halide perovskites. Similarly, various ML works on screening different materials (other than halide perovskites) for photocatalytic and photoelectrochemical water splitting have already appeared in the literature in recent years.^177,178^ Therefore, we expect to see more research covering ML applications for halide perovskites for solar water splitting as well as photocatalytic and photoelectrochemical CO_2_ reduction. Water-resistant halide perovskites can provide improved solar conversion efficiency in CO_2_ reduction, while DFT/ML can make a significant contribution to finding and understanding these materials. Future of this research field looks promising, especially if we also consider the impact of potential developments in experimental and computational resources, including more efficient ML algorithms and increasing data availability on photocatalytic and photoelectrochemical applications of halide perovskites.
